# Estrogens, Cancer and Immunity

**DOI:** 10.3390/cancers14092265

**Published:** 2022-04-30

**Authors:** Izabela Orzołek, Jan Sobieraj, Joanna Domagała-Kulawik

**Affiliations:** 1 Students’ Research Group ‘Alveolus’, Medical University of Warsaw, 02-097 Warsaw, Poland; s074738@student.wum.edu.pl (I.O.); s077888@student.wum.edu.pl (J.S.); 2 Department of Internal Medicine, Pulmonary Diseases and Allergy Medical University of Warsaw, 02-097 Warsaw, Poland

**Keywords:** estrogens, cancer, immunity

## Abstract

**Simple Summary:**

In this review, we present current knowledge of sex hormones in the process of development of malignant diseases. The impact of sex hormones on immune system is presented. A special account is devoted to estrogens and their role in modification of anticancer immune response.

**Abstract:**

Sex hormones are included in many physiological and pathological pathways. Estrogens belong to steroid hormones active in female sex. Estradiol (E2) is the strongest female sex hormone and, with its receptors, contributes to oncogenesis, cancer progression and response to treatment. In recent years, a role of immunosurveillance and suppression of immune response in malignancy has been well defined, forming the basis for cancer immunotherapy. The interplay of sex hormones with cancer immunity, as well as the response to immune checkpoint inhibitors, is of interest. In this review, we investigate the impact of sex hormones on natural immune response with respect to main active elements in anticancer immune surveillance: dendritic cells, macrophages, lymphocytes and checkpoint molecules. We describe the main sex-dependent tumors and the contribution of estrogen in their progression, response to treatment and especially modulation of anticancer immune response.

## 1. Introduction

Malignant diseases constitute a growing problem worldwide, with increasing incidence and mortality. The main type of malignant neoplasms are solid tumors with principal representatives: cancers. Cancers are the first or second leading cause of death among people younger than 70 years of age in many countries [1]. Breast cancer and lung cancer are the most frequent types, with each yielding more than 2 million new cases yearly [1]. Countless studies on epidemiology, biology and therapeutic options in oncology are ongoing. Recent years have seen the introduction of a novel perspective on two aspects of cancer knowledge. One is altered immunity and immune host defense being a basis of immunotherapy. The second is the role of sex diversity in malignancy. Both topics are wide-ranging and not possible to describe comprehensively. We previously pointed to the specificity of lung disease in women [2].

The aim of this review is to summarize current knowledge on the role of estrogens in carcinogenesis, as well as their reactivity in anticancer immune responses.

## 2. Estrogens: Overview

**Figure 1:**
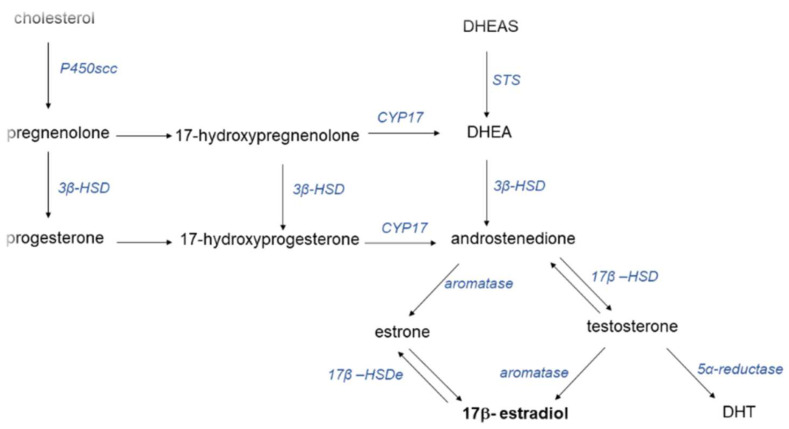
Synthesis of sex hormones. Abbreviations: 3β: hydroxysteroid dehydrogenase; 17β: hydroxysteroid dehydrogenase type 1; CYP17: 17a-hydroxylase; DHT: 5α-dihydrotestosterone; DHEA: dehydroepiandrosterone; DHEAS: dehydroepiandrosterone sulfate; P450scc: cytochrome P450 side-chain cleavage enzyme; STS: steroid sulfatase.

Estrogens are steroid hormones that regulate multiple physiological processes in order to maintain homeostasis [3]. Cholesterol is the precursor of both male and female hormones. The core structure of steroid hormones is formed by a complex of 17 carbon atoms forming a four-ring system. Progestins, androgens and estrogens are sex hormones with different numbers of carbon atoms. There are three biological active estrogens: estrone (E1), 17β-estradiol (E2) and estriol (E3). During life, the predominant female sex hormone is estradiol; during pregnancy, estriol, predominates; and in the postmenopausal period, estron predominates. Estriol has the lowest activity, whereas E2 is as much as 10 times stronger that E1. The levels of sex hormones depend on age and hormonal stage. The range of the E2 serum level is wide, fluctuating between 80 and 800 pM. During pregnancy, this level is elevated to as much as 150 nM. Post-menopause, it ranges between 40 and 120 pM [4]. Interestingly, the level of androgens in women is higher than that of estrogens. Figure 1 presents the process of sex hormone synthesis.

Androgens are precursors of estrogens. The enzyme aromatase (CYP19A1) is responsible for the bioconversion of precursor androgens into estrogens. Aromatase is a member of the cytochrome P450 superfamily. Aromatase is expressed in gonads, endometrium, blood vessels, liver, lung, bone, skin, adipose tissue and brain. Estrogens are synthetized in the ovaries during procreation and, after that, are produced in gynecological and non-gynecological organs. Adipose tissue is a main site of steroid hormones in the body. Primary sites of estrogens include the ovaries, placenta, and breast tissue, although they can also be detected in the skin, bones, brain, liver, lung and adrenal glands [5]. Distribution to tissues is regulated by sex hormone binding protein (SHBG). This protein is produced in the liver. E2 binds with SHBG in 20–40%. 

There are endogenous or exogenous sources of estrogens. Synthetic estrogens are taken as contraceptive or substitutive in hormonal replacement therapy (HRT). Other sources of estrogens include food (phytoestrogens) and metabolites from the environment (xenoestrogens) [6]. Hormonal status and estrogen balance can be evaluated by the measurement of hormones in blood or tissue, as well as the expression of estrogen receptors (ERs) in normal and pathological tissue. These methods can be used in clinical practice [7]. The sex balance during a life depends on age. Aging changes sex hormone levels in males and females in different proportions. Individual hormonal status depends on hormone levels, protein-binding estrogens (like: SHBGs), body mass index (BMI) and receptor activity [8].

Estrogens interact with their receptors. There are two nuclear receptors: estrogen receptor α (ERα, ER1) and estrogen receptor β (ERβ, ER2). ERα and ERβ are nuclear receptors encoded by genes: *ESR1* and *ERS2*, respectively [9,10].

Other receptors active in estrogen function include membrane receptor (mER) and G protein-coupled receptor 1 (GPER1) [3,5,11,12]. GPER1 binds E2 with high affinity. GPER1 is expressed in many organs. Steroid hormones bind to this receptor with lower affinity than to ERs. E2 is a main ligand to GPER1 (Ki, approximately 6 nM) [3].

Estrogen receptors belong to the family of transcription factors and act by genomic (gene transcription) and nongenomic pathways. ERs are widely distributed in the body, but the distribution and action of the two types of ERs in organs and tissues differ [5]. ERα occurs in the female reproductive system, whereas ERβ occurs in the prostate, colon, cardiovascular, and central nervous systems; both ERs are found in skeletal muscle [5,11,12]. ERα and ERβ are, in some respects, antagonists [5]. There are three isoforms of Erα. The isoform wild-type ERβ1 and four additional forms with different biological roles are known: ERβ2, ERβ3, ERβ4 and ERβ5. ERs are also differently expressed in healthy vs. cancer tissue [10]. The function of ERs in malignancy has been recognized to the greatest extent in breast cancer. Studies showed that, in general, whereas ERα is involved in tumor progression, ERβ has antiproliferative properties [9,10]. However, many factors are capable of modifying this function. On a molecular level, ERα and ERβ present antagonism depending on genetics, cell type and the origins of estrogens [10].

Sex hormones act by the following signaling pathways:

- Direct genomic pathway binding to ERs in cytoplasm;
- Indirect genomic signaling;
- Non-genomic signaling (for example, GPER1) [10,11].

ERs are also expressed in immune cells, including macrophages, dendritic cells and lymphocytes, wherein ERα is expressed rather on T cells and ERβ is expressed on B cells. The presence and expression of ERs determine the effect of estrogens on immune response [4,13].

## 3. Estrogens and Cancer

E2 not only mediates biological reactions and preserves homeostasis in normal conditions but is also responsible for the development and spread of malignant tumors. Estrogens influence the progression of a range of hormonally responsive tumors. These include breast, prostate, gynecologic, lung, colorectal cancer and other tumors [14].

### 3.1. Breast Cancer

As much as 80% of breast carcinomas have been found to be ER-positive and plausibly influenced by circulating estrogens [14]. The figures vary from 70 to 80%. Moreover, they are linked to the cut-off. In Europe, tumors with a marker of more than 10% are considered positive, whereas in the United States, the cut-off is 1%. Receptor ERα dominates; ER-positive cancers refer to these receptors, and E2 function is mediated by them. Furthermore, the local synthesis of estrogens in breast cancer tissue may result in eight-fold higher intratissular concentration of the sex hormone compared to plasma levels [15]. It has been confirmed that E2 circulating in the plasma promotes the expression of multiple estrogen-responsive genes included in the process of oncogenesis [16,17,18,19,20]. Although estrogens affect the progression of breast cancers, overall survival (OS) is improved in patients with ER-positive tumours compared to ERα-negative patients. This might be the result of the availability of adjuvant endocrine therapies for ER-positive patients [21,22]. ERβ is expressed in normal mammary glands. In breast cancers, however, it is lost. ERβ agonists in ERα-positive cancers have antiproliferative properties [23]. New data prove that ERβ inhibits breast cancer cell migration and invasion via Claudin-6 (CLDN6)-mediated autophagy. When E2 interacts with ERβ, the expression of CLDN6 is upregulated. This chain reaction leads to beclin1 expression, which is crucial for autophagy. As a result, inhibition of breast cancer metastasis is observed [24]. Human epidermal receptor 2 (HER2) belongs to the tyrosine kinase receptor family and, together with ERs and PRs, is a molecular marker, playing a role in targeted therapy. A proportion of 10% of hormone-receptor-positive breast cancers are HER2+. It was shown that HER2+ hormone-positive cancers are resistant to hormonal therapy [25].

Most results from prospective observational studies and randomized controlled trials show that the use of HRT increases breast cancer incidence [26,27,28]. Contraception was observed to slightly increase breast cancer risk [29]. This effect depends on the origin of progesterone used in HRT. It was also found that analysis of mammographic density is useful in the assessment of breast cancer risk connected with HRT. High density was found to be a marker of high risk [30].

Triple-negative breast cancer (TNBC) is an exceptionally aggressive type of breast cancer that remains the most challenging subtype to treat [31,32]. It is characterized by the absence of estrogen and progesterone receptors, as well as no overexpression or amplification of HER2 [33]. TNBC accounts for 15–20% of all breast cancer cases and usually displays higher grade upon diagnosis, high mitotic index, early recurrence and frequent metastasis [33,34]. After recognizing TNBC subtype, patients have a median OS of 12 to 18 months. Therefore, alternative therapies and newer drugs are urgently required [34].

### 3.2. Ovarian Cancer

Ovarian cancer is known as a hormone-dependent malignant tumor. For many years. estrogens were suspected as a main risk factor. Recently, it was show in many studies that hormonal dependence in ovarian cancer is complex [35]. In vitro experiments showed that E2 can stimulate the growth of ovarian cancer cell lines [36,37]. Additionally, the role of estrone in ovarian cell proliferation was highlighted [35]. The high expression of estrogens in ovaries has no simple translation into carcinogenesis., and There are differences in ER expression depending on histological type [35]. Although both receptor types, ERα and Erβ, can be found in ovarian tumor tissue, the overexpression of ERα is highly distinguishable [38,39]. ERβ was found to have a protective function, as in breast cancer [23]. ERβ agonists may be included in therapeutic strategies. ERα was found to be expressed in higher levels (70–100%) than ERβ (30–70%) [35]. The results implied that unconjugated E2 was positively associated with non-serous ovarian cancer incidence [40]. Progesterone receptors are also involved in hormonal balance in ovarian cancer, with a protective role [35,41]. Taken together, hormonal balance during a life has a role in ovarian cancer development; however, many more pathways are involved in this process and are targets for therapy [42,43]. 

### 3.3. Prostate Cancer

In the prostate, androgen receptors (ARs) function as gene regulators that promote cell survival. It is well known that the ERβ is also present in prostate tissue, yet its role concerning the suppression or enhancement of cancer development remains undetermined [14,44,45]. It was demonstrated in animal models that ERα is upregulated in high-grade prostatic intraepithelial neoplasia (HGPIN). Researchers suggest that it may mediate the carcinogenic effects of E2 in atypical tissue [46,47]. ERβ is partially lost in HGPIN, suggesting its tumor-suppressing ability. The discovery of potent anti-prostate cancer activity of estrogen–artemisinin hybrids has encouraged scientists to further investigate the oncogenic properties of estrogens [48,49].

SERMs (selective estrogen receptor modulators) are successfully used in the treatment of breast cancer. Considering the presence of ERs and their modulating activity towards prostate cancer (PCa) progression, it has been proposed to use SERMs as therapeutic agents in PCa [50]. Zazzo et al. (2018) claim that tamoxifen, toremifene and raloxifene can inhibit the growth of PCa in mouse models and cell cultures. Nonetheless, clinical trials in which high-dose SERMs were used demonstrated poor effectiveness [51]. However, Lafront et al. (2020) stated that widely used PCa models are not suitable to study ERs and that alternative models are needed to determine the roles of the estrogen signaling pathway in PCa [50]. Recently, it was proposed that immunotherapy combined with the use of SERMs can regulate the tumor microenvironment and thus become an option for future adjunctive therapy in PCa. More clinical trials based on immunotherapy and SERMs are necessary [52].

### 3.4. Colorectal Carcinoma

ERβ plays a crucial role in sustaining proper epithelial architecture in healthy colonic tissue. Moreover, it has an impact on gastrointestinal physiology and can mediate various immune responses [53,54]. It has been documented that a decrease in mRNA expression endorses inflammatory processes via higher gut permeability, thus increasing the risk of colorectal carcinoma [55]. Researchers suggest that Erβ may protect colorectal tissue from oncogenic growth, as its expression significantly plummets during cancer progression [56,57]. The amount of available data remains limited due to a shortage of studies on the differential expression of ERs in cancerous and normal colonic epithelia [53]. The early identification of disease is essential; thus, scientists have targeted Erβ expression as a possible value in colorectal cancer prevention [58].

### 3.5. Lung Cancer

As mentioned above, lung cancer is the best example of a common solid tumor. There are different histological types of lung cancer. The two main types are small cell lung cancer (SCLC) and non-small cell lung cancer (NSCLC). NSCLC is divided into three major subtypes: adenocarcinoma, squamous cell carcinoma and large cell carcinoma. The morphological characteristics, as well as molecular alterations, of lung cancer are well defined [59]. This cancer affects patients of both sexes, with a higher incidence in men. Estrogen receptors (ERα and ERβ) are found in healthy lung tissue: muscles, epithelial bronchial cells and non-epithelial cells (immune cells). ERs are involved in lung development. Both estrogen receptors, ERα and ERβ, are present in NSCLC cell lines. A high expression of ERs was reported in lung adenocarcinoma [60,61]. Aromatase is also present in NSCLC tissue. ERβ is capable of activating full estrogen response, and this receptor is a main player in sex-related cancer progression [62,63]. Its function depends on ERβ isoforms. It was found that ERβ1 expression is a negative prognostic factor [23]. ERβs are expressed both in the cytoplasm and in the nucleus, mediating response through non-genomic and genomic mechanisms, respectively [64]. ERβs are also found in the mitochondria of cancer cells. Studies have shown that mitochondrial ERβ can increase the transcription of cytochrome oxidase subunits, increase respiration capacity and elevate antioxidant activity, thus stimulating mitochondrial metabolism to increase energy levels [65,66,67]. Mitochondrial ERβ might be involved in the inhibition of apoptosis via suppression of Bax activation and cytochrome c release [65,66]. Decreased mRNA ERβ levels may be caused by a negative feedback regulation after activation of the estrogen pathway [62]. Although the expression of ERs in adenocarcinoma is similar between genders, it has been reported that lung adenocarcinoma cell lines from females are more responsive to E2 compared to male cell lines. Thus, the difference in response to E2 may not be due to differences in ER expression but due to activity of some critical components of the ER signaling pathways [60]. ERβ increases C-X-C chemokine receptor type 4 (CXCR4) expression, which plays a significant role in migration and metastasis [68]. Another mechanism by which ERβ can promote progression is by inducing upregulation of MMP-2 levels, which contribute to cancer cell migration, invasion and metastasis. Additionally, the expression of ERβ was found to be weaker in primary tumors in comparison with metastatic lymph node tissues from the same patients [69]. Estrogen also upregulates osteopontin expression and secretion, which promotes lung cancer cell metastasis via the MEK/ERK pathway [70].

Estrogen can be produced locally in lung tissue, mainly by aromatase, which converts androgen into estrogen [64]. The intratumoral E2 concentration in NSCLC positively correlates with the level of aromatase expression [61]. Reports show that high aromatase concentration is a poor prognostic factor in NSCLC for both genders. [71] Another study indicates that higher aromatase expression in lung cancer tissue corelated with shorter survival, yet only in females. An advantage in prolonged overall survival was especially pronounced for women 65 years of age and older with low aromatase levels [72]. It has also been reported that high aromatase levels combined with high ERβ expression worsens the survival rate significantly more than each marker independently [73].

Another important pathway is the relationship between epidermal growth factor receptor (EGFR) activity and estrogens in NSCLC. EGFR is mutated in high frequency in adenocarcinoma and acts in carcinogenesis [74]. Estrogens have been found to stimulate EGFR activity and vice-versa [75]. EGFR signaling contributes to stimulation of aromatase. A high EGFR mutation rate correlates with ERβ expression. Interestingly, a negative relation between ERβ expression and the effect of tyrosine kinase inhibitor (TKI) treatment was reported [76]. The question arises: could expression of Erβ be an additional biomarker for targeted therapy?

Regarding the association of HRT use and risk of developing lung cancer, the findings are conflicting; some studies report a reduced risk of lung cancer [77,78], whereas others show an increased risk [79]. A post hoc analysis of a randomized controlled trial showed that although treatment with estrogen (0.62 mg) and progestin (2.5 mg) did not increase the incidence of lung cancer, the mortality was higher, especially from NSCLC. Treatment with HRT also contributed to distant metastasis and less differentiated tumors [80].

### 3.6. Other Malignancies: Examples

Recently, it has been concluded that gliomas and, more specifically, astrocytomas are hormone-sensitive tumors that could be influenced by estrogens present in brain [81]. The most severe and aggressive form of astrocytoma, glioblastoma, was observed to present lower expression of ERs as the degree of malignancy increases, suggesting that ERs play a crucial role in the development and possibly the treatment of tumors [82].

Although tumors of hematopoietic and lymphoid tissues are not sex-related diseases, there are some discrepancies in their incidence between genders. It has been reported that peripheral blood mononuclear cells (PBMCs) express both estrogen receptor subtypes in subjects with chronic lymphocytic leukemia (CLL) [83], whereas, for instance, in acute myeloid leukemia (AML), ERβ was found to be the most abundant subtype. Concerning lymphomas, studies show that ERβ in murine models of Burkitt, Hodgkin [84] and mantle cell lymphomas [85] is predominant, whereas ERα expression is weaker.

## 4. Sex Hormones and Immunity

**Figure 2:**
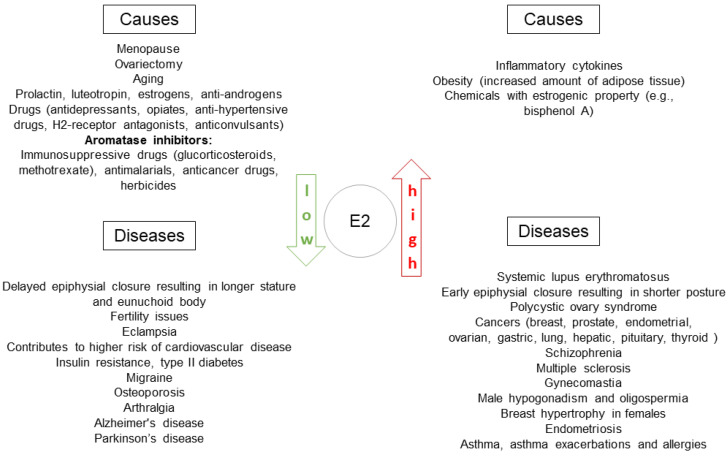
Diseases related to estrogen balance with observed prevalence among women. E2: estradiol.

Sex hormones and their receptors take part in regulation of immune status in health and disease. Clinical data, basic research and experimental studies provide evidence of the important role of estrogens in immunity. Clinical observations show that there is a prevalence of some diseases with immune background in women (Figure 2). The best example are autoimmune diseases.

An improvement of some diseases during pregnancy is observed (for example, rheumatoid arthritis). The effect of rituximab is more pronounced in women than in men [86]. HRT is capable of reversing the influence of aging on immunity. An increase in B-cell and T-cell count and a decrease in inflammatory cytokines was observed [29].

The direct impact of sex hormones on the immune system is known. In general, testosterone is related to an immunosuppressive effect, whereas estrogens correlate with immunoenhancement. Dendritic cells (DCs), macrophages, natural killer T-cells (NKT), T cells and B-cells are sex-hormone-sensitive immune cells. The effects of estrogens include the elevation of phagocytic activity of macrophages; antigen-presenting function of DCs; differentiation in the Th2 direction; higher T-cell and B-cell contributions but lower eosinophils contribution; and cytotoxic CD8 cells, NK and ILC-2 cells [86,87]. E2 stimulates IFNγ and TNFα. However, there is no unequivocal translation between estrogens and immune status—it is more complex and labile, depending, among other factors, on hormone level and receptors expression. High E2 concentrations inhibits TNF, IL-1B an IL-6, whereas lower concentrations induce the opposite effect [88].

Some genes on chromosome X are included in the regulation of immunity [87]. Some interleukins, toll-like receptors and transcription factors are listed.

ERs are involved in the regulation of the immune system by estrogens. It was found that ERs are encoded by *ESR1* and *ESR2* genes for ERα and ERβ, respectively [13]. Expression of these genes was found in human immune blood cells, as well as in progenitors of immune cells. The expression of *ESR1* and *ESR2* RNA is high in human B cells but lower in T cells and DCs. *ESR1* expression changes during differentiation of DCs. Estrogens are involved in the regulation of the genetics of ERs.

Epigenetic and environmental factors can modulate this sex-dependent immune phenotype. Sex hormones affect the immune system (Table 1); thus, immunological responses differ in males and females [89]. There are differences in innate and adaptive immune responses in pre- and postmenopausal women. There is a reduction in B and T cells, a decline in IFNγ levels and an increase in proinflammatory cytokines, such as IL-1, IL-6 and TNFα, after menopause [90]. Postmenopausal women treated with HRT had more B cells, higher T-cell proliferation and higher levels of TNFα [91], suggesting that HRT may reverse postmenopausal changes in the immune system resulting from cessation of sex hormones.

## 5. Cancer Immunity

**Figure 3:**
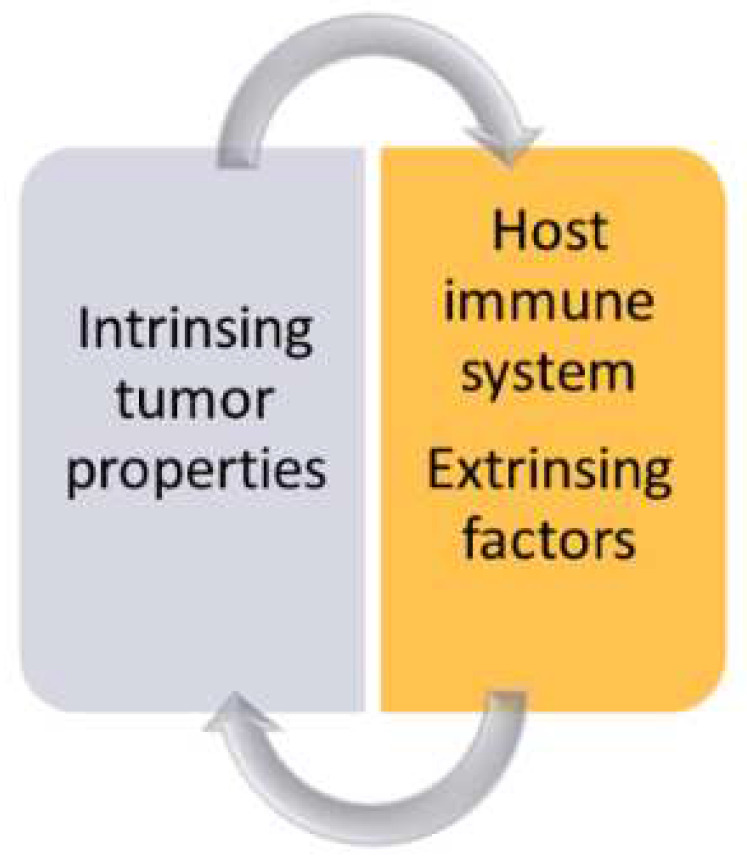
Immune response in cancer depends on tumor characteristics such as molecular alterations and the status of host immunity modified by environmental factors, such as, among others, sex hormones.

The success of immunotherapy with checkpoint inhibitors (ICIs) of solid tumors has introduced a new perception of cancer interplay with the tumor environment (TME). The role of immune response in cancer has been known for years. Many methods of cancer immunotherapy before ICIs were found to be more or less effective but with no established order among recommended therapies in oncology. Finally, ICIs found their place in the first-line of treatment of patients with advanced tumors [92]. However, the response to treatment is highly individual, with prolonged survival only for some patients. To date, there are no predictive markers for therapy with ICIs apart from expression of PL-L1 on tumor cells. The response to ICIs depends on tumor biology, as well as host immune status (Figure 3).

**Figure 4:**
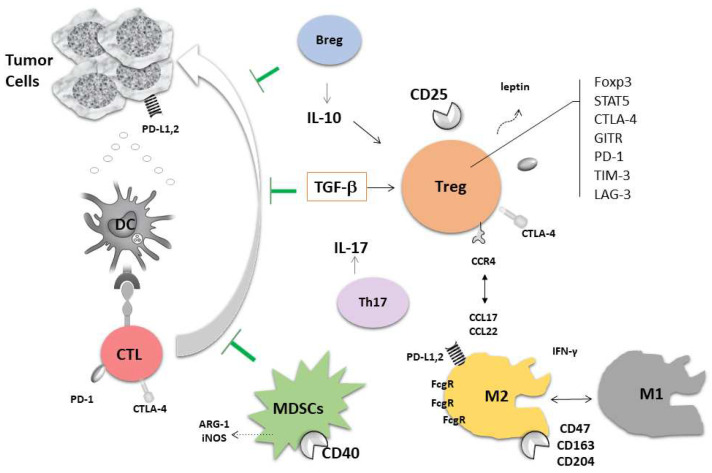
Main elements of regulation of immune response and the pathways of immunosuppression in the tumor environment. The anticancer activity of cytotoxic T cells (CTLs) is inhibited (˫) by complex and cooperative cells and mediators. Their activity is plastic and depends on local conditions. Abbreviations: Breg: regulatory B cell; DC: dendritic cell; Treg: regulatory T cell; Foxp3, STAT5, CTLA-4, GITR, PD-1, TIM-3, LAG-3: active molecules on/in Treg; MDSCs: myeloid-derived suppressor cells; M: macrophages; PD-1: programmed death-1; L: ligand; CTLA-4; cytotoxic T cell antigen-4; INFγ: interferon γ; TGF-β; transforming growth factor β.

The term “immunoscoring” illustrates the role of TME in susceptibility to ICIs. Galon et al. described the methods of immunoscoring evaluation in colon cancer [93]. The results of many other studies confirmed the necessity of assessment of the character of immune infiltration in cancer tissue and surrounding “normal” tissue. The network of immune reactions in the human body in the course of malignant disease is highly complicated but similar, irrespectively of the origin of the tumor. In general, the host anticancer immune response is suppressed by many factors; these pathways are well-recognized to date (Figure 4) [94,95,96].

The TME of tumors with a high resection rate, such as melanoma, colon carcinoma and breast cancer, have been well-characterized [97,98], whereas, for example, in NSCLC, the resection rate is as low as 30% and other methods of TME evaluation are needed [99,100]. Recently, it was confirmed that “hot” tumors characterized by the presence of rich immune infiltration respond better to immunotherapy. These inflamed ‘hot’ tumors are rich in pro- and anticancer immune cells, mediators and checkpoint-positive cells. This is a TME in which the immune game ‘goes on’ [101]. As programmed death-1 (PD-1) is a molecule expressed on immune cells, mainly lymphocytes, PD-L1 is a ligand for it. PD-L1 is overexpressed on cancer cells. The ligation of PD-1 with PD-L1 causes a suppressor signal on immune cells [102]. PD-L1 has been detected in primary tumor cells, in metastases and liquid biopsy. Extracellular vesicles and exosomes are capable of bringing tumor “signature” on the status of PD-L1 molecule [103]. Cancer stem cells (CSC), also called ‘cells initiating tumor’ may cause resistance to systemic therapies and contribute to the modification of immune response in the TME. Our studies confirmed this capability in NSCLC [100]. CSCs were identified in the blood and lymph nodes (LNs)by flow cytometry.

NSCLC is a good example of the role of the TME in cancer prognosis. In a meta-analysis, Soo et al. presented the significance of specific cells in the TME [91]. The analysis summarized 96 individual studies representing 21,752 cases. It was confirmed that DCs, NK cells, M1 macrophages, CD8+ T cells and B cells in the tumor and stroma are associated with a good prognosis, whereas stromal M2 macrophages, regulatory T cells (Tregs) and PD-L1 overexpression are associated with an unfavorable prognosis in NSCLC. There is evidence that cancer immunity and the response to immunotherapy depend on the genetic background. Tumors with a high tumor mutational burden (TMB) are found to better respond to ICIs than silent tumors. TMB was recently proposed as an important predictive factor in addition to PD-L1 expression [104,105]. Rosenthal et al. showed that the relationship between tumor antigens and the activation of the immune system is highly individual not only between subjects but also across the tumor [106]. The relationship between immunotherapy and treatable driver molecular alterations is of interest. It was shown that patients with EGFR mutations did not benefit from ICIs in overall survival vs. chemotherapy [104], as confirmed by other studies.

There are numerous mechanisms of resistance to ICIs: lack of immunogenicity, enhanced T-cell exclusion, lack of response to IFNγ, an increased prevalence of immunosuppression in the TME, upregulation of other immunosuppressive receptors on T cells: cytotoxic T cell antigen- 4 (CTLA-4), T-cell immunoglobulin and mucin domain-containing molecule 3 (TIM3); lymphocyte activation gene 3 (LAG-3); and V-domain Ig suppressor of T-cell activation (VISTA), low neoantigen production [107].

There are some important processes other than the strict immune response in the interplay between cancer and the host. These are autophagy and epithelial–mesenchymal transition (EMT). Autophagy serves the preservation of cell homeostasis. It is a biological process of intracellular degradation, recycling and removing cell products. Autophagy may be induced by extrinsic factors, such as stress. In malignancy, the role of autophagy is complex; it may contribute to cancer cell survival and tumor progression or to cell death. Autophagy and apoptosis are associated with what is important in the context of anticancer therapy: autophagy protects cancer cells. The role of EMT in cancer progression is to facilitate the ability to spread and increase the metastatic potential. This is a process in which epithelial features of cancer cells change into mesenchymal-like phenotypes [108].

## 6. Sex Hormones and Cancer Immunity: Therapeutic Implications

### 6.1. Estrogens and TME

In the analysis of the relationship between sex hormones and cancer immunity, some aspects need to be considered to achieve a broad overview:

- A natural response in women;
- Fluctuation of hormonal balance during life;
- Immunoaging;
- Direct impact of estrogens on immune cells and their progenitors;
- Influence of hormones given in HRT;
- Interplay of cancer immune response and natural hormonal status.

Thanks to numerous studies, recognition of this difficult topic has been achieved. Sex hormones, i.e., estrogens, have an impact on almost all known directions of anticancer immune response. In general, estrogens contribute to immunosuppression, thus helping malignant tumors to escape from immunosurveillance.

The TME consists of heterogenous components, such as cancer-associated fibroblasts (CAFs), tumor-associated macrophages (TAMs), myeloid-derived suppressor cells (MDSCs), T and B cells, natural killer cells (NK), endothelial cells and the matrix [109]. Thus, cancer progression is accomplished by the interplay between cancer cells, non-neoplastic cells comprising TME, as well as immune cells [110]. Of the last DCs, macrophages, NK cells, T lymphocytes and Tregs with expression of checkpoint molecules, need special attention.

DCs are crucial in antigen presentation and are important players in anticancer responses. The population of DCs is not uniform [111]. ERs, which are expressed on DCs, as well as nuclear ERα, were described to play a role in the regulation of DC function [112]. There is no strict direction of estrogen-related DC function in cancer; however, it seems dependent on local regulation in the TME. Similarly, the data on Tregs are confusing; what could be expected as a population of Tregs is not uniform and depends on local circumstances and the expression of checkpoint molecules [113].

An estrogen-rich environment might contribute to an enhanced differentiation of pulmonary macrophages into M2 phenotypes [114]. In studies with ovariectomized mice, it was shown that the number of M2 macrophages of those mice declined, whereas mice with E2 supplementation had higher IL-4 levels, leading to an enhanced M2 polarization. A higher expression of ERα is corelated with higher levels of infiltrated TAM in the TME. ERα also activates M2 polarization and increases production of MMP-9 by macrophages, which might result in increased NSCLC invasion [115]. TAM also express aromatase, which suggests local release of estrogen in the tumor microenvironment [110,116]. Estrogen has been shown to enhance the production of VEGF by pulmonary macrophages in the lungs of mice exposed to a tobacco carcinogen [117].

Estrogen receptors were found to influence the process of autophagy in cancer tissue and in the TME [108]. This process also concerns immune cells. The pathways of autophagy were found to be mediated by ERβ in malignant tumors of the colon and lung, as well as in melanoma and non-Hodgkin lymphoma [110]. Autophagy could be a target for anticancer therapy; however, this action should be delicate, as the process is ambiguous.

The role of the PD-1/PD-L1 axis in suppression of anticancer response is well-known [118]. Upregulated expresion of PD-1/ PD-L1 has been shown to be increased by estrogens [119]. The results of experimental studies led to a conclusion that E2 influences the expression of PD-1 on Tregs [120]. In experimental models, E2-induced PD-1 and Tregs were shown to be involved in regulatory–suppressive immune response, with PD-1, Tregs, IL-17 and forkhead box P3 (FOXP3) activity [121]. However, observational studies do not indicate any significant differences in PD-L1 expression between men and women [122], which was confirmed in some of our studies. We explored immune cell phenotypes and expression of immune checkpoints of immune cells derived from the blood and TME of lung cancer patients [123]. When analyzing differences between sex, we did not find spectacular results. The proportion of PD-1+ and PD-L1+ cells in metastatic LNs was similar in men and women. The proportion of PD-1+ T cells in bronchoalveolar lavage fluid (BALF) from the lung affected by cancer did not differ between men and women. However, we found some differences in the maturation of lymphocytes: we found a higher proportion of central memory CD4+ and CD8+ cells in women but a lower proportion of effector cells in metastatic LNs when compared with men; these differences were significant.

The majority of malignant tumors develop in the elderly. However, aging is not the only reason for the appearance of cancers. In the breast, ERα cancers occur during menopause, and with age there, is an increased risk of chromosomal alterations that accumulate over time. Similarly, a decrease in physical exercise can lead to overweight and changes in steroid hormone balance. It is important to remember that the development of cancer is multifactorial.

Some remarks need to be made in relation to changes of the immune system in older age. It has been reported that immunoaging generally reflects chronic inflammation [90]. Innate immune cells are less functional but have more proinflammatory properties with age. The expression of the CD62L activation marker on monocytes is higher in females than in men. The repertoire of T cells changes to more regulatory phenotype. There is an elevated secretion of cytokines: IL-6, TNF and IL-1β are more pronounced in postmenopausal women than in older men. Additionally, cancer immunosurveillance, e.g., the activity of NK cells, is more effective in women. In the analysis of the impact of age on immune status, hormonal status should be considered: the time of menopause, HRT and, eventually, the level of sex hormones in the blood. As many stimuli influence the secretion of inflammatory mediators in advanced age (e.g., oxidative stress, telomerase shortness and epigenetic alterations), a descriptive name has been suggested: senescence-associated secretory phenotype (SASP) [124]. Inflammation is prevalent in older men, whereas higher cellular (NK and CD4 cells) responses predominate among women [89].

### 6.2. Breast Cancer and Immunity

An instructive lesson on the subject of the impact of estrogens on cancer immunity can be drawn from studies in breast cancer. In general, breast cancers are silent, with low mutational activity, modest immune infiltration by lymphocytes (tumor-infiltrating lymphocytes, TILs) and low PD-L1 expression [125]. Other features of breast cancer, apart from low TILs, include a lack of tumor antigenicity, dysregulation of the WNT-β-catenin pathway, the loss of phosphatase and tensin homolog (PTEN) and p53 and deletional mutations in the JAK1/2-STAT signaling pathway [126]. The special and frequent forms are hormone-positive (HR+) tumors, which account for about 70–80% of cases subtypes, are typically “cold”, with low response to ICIs. The immune infiltration in these tumors presents immunosuppressive properties [125]. As anti-PD-L1 and anti-PD-1 therapies have low effectiveness, the other checkpoints promise to be the better targets for immunotherapy. It has been reported that a kind of lymphocyte infiltration depends on ER status in breast cancer samples. ER-positive samples showed lower infiltration by B cells, cytotoxic T cells and Th1 cells than ER-negative samples [127].

The expression of the PD-1 pathway correlates with TILs [128]. Wherein TILs are not uniform, our study showed that lymphocyte infiltration is different in composition, density and localization in the tumor and surrounding tissue. There was a different maturation status of lymphocytes; the memory T and B cells dominate in the TME [128], which is in agreement with our results in lung cancer [111]. In a recent study, Terranova et al. demonstrated that the exhausted CD8 cell phenotype, CD8+/PD-1+/CTLA-4+, and depletion of CD4+/FOXP3+/CTLA-4+ implicate a better response to ICIs in HR+ breast cancer [126]. Histone deacetylase inhibitors (HDACis), such as vorinostat, are epigenetic factors capable of modifying response to hormone therapy in HR+ breast cancer, influencing autophagy and apoptosis [108,126]. HDACis reduce Tregs, increase expression of PD-L1 and induce CD8 T cells [129].

TNBC were shown to be responders to ICIs, thanks to high TMB, rich TILs and high PD-L1 expression [130]. ICIs (atezolizumab and pembrolizumab) are recommended in advanced/metastatic TNBC with detectable expression of PD-L1 [131]. A comprehensive review by Stovgaard et al. presents characteristics of the immune profile of the TNBC in detail [132].

### 6.3. Estrogens and Immunotherapy

Results of treatment using immunotherapy could help in understanding the role of sex hormones in immune regulation of cancer progression, which can be exemplified by NSCLC. In general, the prognosis in lung cancer is better among women than men. However, with regard to immunotherapy no evident benefit of ICIs among women is observed [133]. Mechanisms of resistance to immunotherapy in women are suspected. One of them is low TMB [134], which could be expected, as tobacco smoking is related to a high rate of molecular alterations [135] and smoking history among women is much lower than in the male sex. Adenocarcinoma with oncogenic addiction is dominant in women; the relation of EGFR and ERβ was described above. According to a meta-analysis of 22 studies, the overall overexpression of ERβ in malignant epithelial cells is associated with poorer overall survival (OS) [62,71]. However, considering localizations of ERβ, high expression of cytoplasmic ERβ is negatively associated with OS, whereas increased nuclear ERβ had no significant impact on OS [62]. Conversely, levels of ERβ mRNA have no predictive value in lung adenocarcinoma overall survival rates [62,136]. The level of ERβ expression was found to be significantly correlated with tumor size, lymph node metastasis, clinical stage and tumor differentiation [137]. Recent research showed that ERβ can induce radioresistance in NSCLC cells [138]. Estrogen is involved in inducing chemoresistance; E2 can induce chemoresistance to cisplatin with association to both ERα and ERβ. Studies have shown that ERα-positive and ERβ-negative tumors promoted E2-induced chemoresistance, whereas ERβ-positive and ERα-negative NSCLC cells were more responsive to cisplatin [139]. Cytoplasmic ERβ has been found to be involved in the development of resistance to EGFR-TKIs in patients with lung adenocarcinoma and relevant EGFR mutations [76]. Downregulation of ERβ may sensitize NSCLC to EGRF- tyrosine kinase inhibitors (TKIs); thus, anti-ERβ treatment may reverse resistance [140].

Data on the influence of ERα on survival are inconsistent. The expression of ERα mRNA and ERα-positive tumors has been reported to be a negative prognostic biomarker for NSCLC for both men and women [141,142]. Conversely, another study showed that patients with ERα-positive tumors had longer median survivals than patients with Erα-negative tumors (10.8 vs. 6.4 months, respectively) [143]. The expression of ERα in NSCLC has also been reported to be a negative prognostic factor for treatment outcomes in patients who received radiotherapy for NSCLC [141]. It has been shown that E2 and TNFα are engaged in uncharacteristic activation of ERα-expressing human lung adenocarcinoma cells, driving a transcriptomic change that results in cisplatin tolerance, cell migration and worsened prognosis [144]. Furthermore, is has been shown that estrogen-related SNPs influence cancer prognosis; a variant of the *ESR1-07* SNP was associated with increased tumor ERα mRNA levels and poorer lung cancer prognosis [142]. According to another study the incidence of recurrence was significantly higher in patients with ERα-positive adenocarcinomas in pT1a stage than those with ERα-negative tumors [145].

Some positive results in cancer treatment could be achieved by modifying the influence of sex hormones on tumorigenesis. Aromatase contributes to the production of estradiol in tumors, with high intratumor expression (for example, in NSCLC). Reports show that high aromatase concentration is a poor prognostic factor in NSCLC for both genders [71]. Another study indicated that higher aromatase expression in lung cancer tissue was corelated with shorter survival, yet only in females. An advantage in prolonged overall survival was especially pronounced for women 65 years of age and older with low aromatase levels [72]. It has also been reported that high aromatase levels combined with high ERβ expression worsens the survival rate significantly more than each marker independently [73].

Exosomes are small extracellular vesicles (EVs) originating from cells bearing important molecules capable of reflecting cell signature. Tumor-derived exosomes (TEXs) were found to play an important role in cancer progression. TEXs are able to modify the immune environment of malignant tumors [146,147]. The presence of PD-L1 in EVs is responsible for modulating immune response in many tumors, as well as the resistance to ICIs [148]. The isoform of aromatase-ARO1 was found in TEXs of patients with ovarian cancer, and the ability of ARO+ TEXs to inhibit CD4 and CD8 cells was described by Czystowska-Kuzmicz et al. [149]. Aromatase is a good target for therapy. The inhibitors of aromatase are used in breast cancer and have been investigated in NSCLC with fulvestrant (a selective antagonist of estrogen receptors). The regimens of consolidation therapy include inhibitors of estrogen receptors and aromatase inhibitors with classical targeted therapies [7]. The addition of antiestrogen therapy to classical approaches has achieved promising results [119,150]. One possible pathomechanism is influence on EMT signaling. One of possible explanations is that antiestrogen therapy influences EMT signaling [88]. Estrogens are responsible for resistance to treatment by EMT induction [88].

## 7. Conclusions

**Figure 5:**
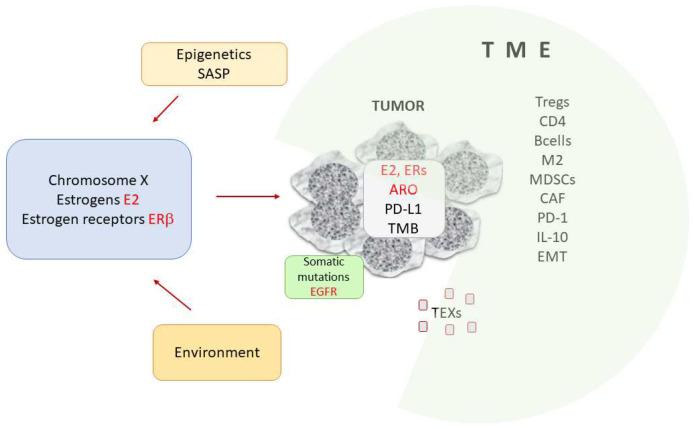
Simplified summary of the importance of estrogens in tumor immunity. The nature of the tumor environment (TME) is crucial in cancer progression. Many cells (Tregs, Bregs, CD4 and CD8 cells, MDSCs, DCs and cells with PD-1 expression), EMTs and mediators contribute to the immunosuppressive function of the TME. Estrogens, estrogen receptors and enzymes (aromatases) are capable of modifying immune anticancer response. Hormonal balance depends on genetic factors connected with the X chromosome, epigenetics and the environment. Estradiol and estrogen receptor beta are the main players in systemic and local regulation of carcinogenesis, tumor progression and modulation of immunity. Abbreviations: ARO: aromatase; CAF: cancer-associated fibroblast; E2-17β: estradiol; EGFR: epidermal growth factor receptor; ERs: estrogen receptors: EMT: epithelial–mesenchymal transition; M2- type 2 macrophages, MDSCs: myeloid-derived suppressor cells; PD-1 – programmed death, PD-L1: programmed death ligand 1; SASP: senescence-associated secretory phenotype: TEXs: tumor-derived exosomes; TMB: tumor mutational burden; TME: tumor microenvironment, Tregs- regulatory Tcells.

Sex hormones are ubiquitous. The balance of female and male hormones is observed in both sexes. The impact of estrogens on the development and spread of different neoplasms seems to be strong. Estrogens are present and locally produced in many cancers. Estrogen receptors are not without importance in oncogenesis. In this review we summarized that the female hormones engage in modification of immune anticancer response by weakening it. To summarize concluding remarks we present here Figure 5.

Estrogen activity is not simple and unidirectional; there is a complex interplay between hormones, receptors and enzymes. Many signaling pathways are triggered, and sometimes the actions are opposite. The hormonal balance resamples immune reactions; the nature of the environment is capable of modifying and directing processes. Sex hormones are involved in oncogenesis and progression of many malignant tumors, not only those known to be hormone-dependent, such as lung cancer. Immune anticancer response and TME have an undeniable and well-known meaning in cancer development and remain an important target for therapy. The results of many studies show that sex hormones are involved in a plethora of immune reactions. The direction of estrogen activity in TME is connected with suppression of anticancer response. Recently, the use of immunotherapy with immune checkpoint inhibitors was established; however, the biomarkers are widely investigated. Our results, as well as those of other studies, support the value of TME investigation before therapy. Our knowledge on tumor biology and host status could be improved by evaluating hormone concentration, expression of sex hormone receptors and enzyme activity. The standardization of TEX detection may by valuable. Altogether, research to date indicates a very important and interesting direction for future studies, as well as new methods of combined therapies, which have been shown to be more effective than monotherapy. However, many questions remain.

## Figures and Tables

**Table 1:** Impact of sex hormones on elements of immunity: cells and mediators.

Targeted Cells	Influence of Estrogens	Influence of Progesterone	Influence of Androgens	References
Neutrophils	Promote neutrophil infiltration: number, degranulation, elastase release Significantly higher expression of neutrophil-attracting chemokines (CCL20, CXCL5 AND CXCL2) in females	Not defined	Increase in numbers Lower function Initiation of neutrophil production via modulation of G-CSF Suppression of superoxide production and antimicrobial capacity of neutrophils Genetic ablation of ARO diminishes (90%) the proliferative activity of neutrophils and retards their maturation	[4,89]
Monocytes	Increase in the number of chemoattractants for monocytes	Not defined	Negative regulation of monocyte levels Lower monocyte infiltration Decrease in IL-6 and TNF-α production	[4]
Macrophages	Promote phagocytic activity Facilitate resolution phase of inflammation toward M2 phenotype dependent on IL-10 Contribution to tissue remodeling and shortening of proinflammatory state Enhance cytokine secretion Increase expression of TLR4, TLR7 and TLR9	Inhibition of NO production and the release of MPs with proinflammatory and prothrombic properties	Decreased expression of TLR4 Reduced production of IL-1β, IL-6 and TNF-α increases the production of IL-10 TES can diminish NO production induced by the stimulus of LPS	[4,89]
Eosinophils	Lower numbers and mobilization	Progesterone treatment enhances recruitment of eosinophils and induces airway hyperresponsiveness	TES decreases human eosinophil viability and adhesion properties in vitro	[4]
Mast Cells	Serum levels of IgE fluctuate depending on the menstrual cycle phase Estrogen enhances IgE-induced mast cell degranulation and release of histamine	Progesterone diminishes the migration of mast cells and histamine secretion	TES interferes with the production of IL-6 and induces the expression of IL-33	[4]
DCs	Promotes differentiation of DCs from bone marrow precursors and enhances their T-cell stimulatory capacity Activation of antigen presentation	Decrease in the production of proinflammatory cytokines TNF-α and IL-1β by BMDCs	Not defined In vitro, 5α-DHT decreases the production of IL-4, IL-10 and IL-13	[86]
	Lymphocytes			
B	Restrain B-cell lymphopoiesis Enhance activity and antibody production of mature B cells		Androgens negatively regulate B-cell development	[4]
T	Elevated CD4:CD8 ratio High CD8 response Physiological concentrations of E2 promote proliferation of T lymphocytes and production of IFN-γ in vitro - Low E2 concentrations promote Th1-type responses and cell-mediated immunity, upregulate MAPK, T-bet and select miRNAs to increase production of IFNγ by T cells - High E2 concentrations augment Th2-type responses Exogenous E2 enhances the expansion of Treg cell populations in mice and healthy women In vitro: E2 increases the number of Treg cells generated from PBMCs and influences IL-17 production (high doses of E2 decrease IL-17 production by Th17 cells, whereas ovariectomy of female mice increases Th17 cell number and IL-17 production)	Th2 > Th1 Low CD8 response	Androgens negatively regulate T-cell development Low CD8 activity Direction to Th-17 differentiation In vitro: reduction in TNF-α secretion, stimulates production of IL-10 In rat models: androgen deficiency decreases levels of IL-2, IL-6, IL-10, IL-12 and IL-13, whereas TES supplementation restores those levels	[4,29,87,89]
NK	High INFγ and granzyme B production	Increased numbers, apoptosis	Not defined	[89]
Cytokines	↑IL-4, IL-10, TGFβ ↓IL-17 - Low E concentration ↑ IL-1β, IL-6, TNF - High E concentration ↓ IL-1β, IL-6, TNF	↓TNF, IFNγ ↑IL-6 ↑IL-4, IL-5, TGFβ	↓IL-4,TGFβ, IL-10 ↑IL-17	[4,89] [89]

Abbreviations: 5α-DHT—5α-dihydrotestosterone, ARO—aromatase, BMDCs—bone marrow-derived dendritic cells, CCL20, CXCL5 CXCL2—chemokines, DCs—dendritic cells, E217β-estradiol, G-CSF—granulocyte colony-stimulating factor, IFN-γ—interferon gamma, IgE—immunoglobulin E, IL—interleukin, M2—macrophages M2, MAPK—mitogen-activated protein kinase, miRNA—micro RNA, MPs—microparticles, NO—nitric oxide, PBMCs—peripheral blood mononuclear cells, TES—testosterone, TGFβ—transforming growth factorβ, Th-17—lymphocyte Th-17, TLR—toll receptors, TNF-α tumor necrosis factor α, Treg—regulatory T cell. ↑—elevated concentration, ↓—diminished concentration.

## Abbreviations

**Abbreviation**	**Meaning**
17β	hydroxysteroid dehydrogenase type 1
3β	hydroxysteroid dehydrogenase
ARs	androgen receptors
ARO	aromatase
BALF	bronchoalveolar lavage fluid
BMDCs	bone marrow-derived dendritic cells
CSCs	cancer stem cells
CXCR4 C-X-C chemokine receptor type 4	
CYP17	17aα-hydroxylase
CYP19A1	aromatase
DCs	dendritic cells
DHEA	dehydroepiandrosterone
DHEAS	dehydroepiandrosterone sulfate
DHT	5α-dihydrotestosterone
E1	estrone
E2	17β-estradiol
E3	estriol
EGFR	epidermal growth factor receptor
EGFR-TKIs	epidermal growth factor receptor-tyrosine kinase inhibitors
EMT	epithelial-mesenchymal transition
ERs	estrogen receptors
ERα, ER1	estrogen receptor α
ERβ, ER2	estrogen receptor β
ESR1	estrogen receptor alpha gene
ESR2	estrogen receptor beta gene
EVs	extracellular vesicles
G-CSF	granulocyte colony-stimulating factor
GPER1	G protein-coupled estrogen receptor 1
HDACi	histone deacetylase inhibitors
HER2	human epidermal growth factor receptor 2
HGPIN	high-grade prostatic intraepithelial neoplasia
HR+	hormone-positive
HRT	hormonal replacement therapy
ICIs	immune checkpoint inhibitors
IL	interleukin
LAG-3	lymphocyte activation gene 3
LNs	lymph nodes
LPS	lipopolysaccharide
MAPK	mitogen-activated protein kinase
MDSCs	myeloid-derived suppressor cells
MMP-2	matrix metalloproteinase-2
MMP-9	matrix metalloproteinase-9
MPs	microparticles
NO	nitric oxide
NSCLC	non-small cell lung carcinoma
OS	overall survival
P450scc	cytochrome P450 side-chain cleavage enzyme
PBMC	peripheral blood mononuclear cell
PD-L1	programmed death ligand 1
SASP	senescence-associated secretory phenotype
SCLC	small cell lung carcinoma
SHBG	sex hormone-binding protein
SNPs	single-nucleotide polymorphism
STS	steroid sulfatase
TAMs	tumor-associated macrophages
TES	testosterone
TEXs	tumor-derived exosomes
TILs	tumor-infiltrating lymphocytes
TIM3	T-cell immunoglobulin and mucin domain-containing molecule 3
TKIs	tyrosine kinase inhibitors
TMB	tumor mutational burden
TME	tumor microenvironment
TNBC	triple-negative breast cancer
TNFα	tumor necrosis factor α
VEGF	vascular endothelial growth factor
VISTA	V-domain Ig suppressor of T-cell activation
